# Entropy Generation Analysis and Radiated Heat Transfer in MHD (Al_2_O_3_-Cu/Water) Hybrid Nanofluid Flow

**DOI:** 10.3390/mi12080887

**Published:** 2021-07-27

**Authors:** Nabeela Parveen, Muhammad Awais, Saeed Ehsan Awan, Wasim Ullah Khan, Yigang He, Muhammad Yousaf Malik

**Affiliations:** 1 Department of Mathematics, Attock Campus, COMSATS University Islamabad, Attock 43600, Pakistan; nabeela.mpa2016@gmail.com (N.P.); awais@ciit-attock.edu.pk (M.A.); 2 Department of Electrical and Computer Engineering, Attock Campus, COMSATS University Islamabad, Attock 43600, Pakistan; saeed.ehsan@cuiatk.edu.pk; 3 School of Electrical Engineering and Automation, Wuhan University, Wuhan 430072, China; 4 Department of Mathematics, College of Sciences, King Khalid University, Abha 61413, Saudi Arabia; drmymalik@hotmail.com

**Keywords:** hybrid nanofluid, entropy generation, induced magnetic field, convective boundary conditions, thermal radiations, stretching disk

## Abstract

This research concerns the heat transfer and entropy generation analysis in the MHD axisymmetric flow of Al_2_O_3_-Cu/H_2_O hybrid nanofluid. The magnetic induction effect is considered for large magnetic Reynolds number. The influences of thermal radiations, viscous dissipation and convective temperature conditions over flow are studied. The problem is modeled using boundary layer theory, Maxwell’s equations and Fourier’s conduction law along with defined physical factors. Similarity transformations are utilized for model simplification which is analytically solved with the homotopy analysis method. The h-curves up to 20th order for solutions establishes the stability and convergence of the adopted computational method. Rheological impacts of involved parameters on flow variables and entropy generation number are demonstrated via graphs and tables. The study reveals that entropy in system of hybrid nanofluid affected by magnetic induction declines for *β* while it enhances for *Bi*, *R* and λ. Moreover, heat transfer rate elevates for large *Bi* with convective conditions at surface.

## 1. Introduction

MHD boundary layer flows of electrically conducting fluids and heat transfer caused by a stretching sheet have gained immense importance due to their ample applications and significant bearings on several engineering and technological processes. Major applications include heat exchangers, metals’ spinning, and power generators, spinning of fiber, aerodynamic extrusion of plastic sheets, polymer industries, and condensation process of metallic sheets inside cooling glass. The quality of the resulting product greatly depends on the stretching process as well as its rate of cooling. Boundary-layer flow combined with heat transfer and followed by a stretching sheet was primarily proposed by Sakiadis [1]. Crane [2] also investigated flow caused by stretching sheets of plastic material. Boundary layer equations describing air motion due to a plate were solved analytically. Rates of flow and heat transfer were analyzed in terms of coefficients of friction and thermal conductivity, respectively. Since then, many attempts have been made in regard to their application in different areas. The analysis of viscous dissipation, thermal radiations and convective wall conditions in fluid flow has its importance in view of its broad coverage including chemical, mechanical, and aerospace engineering, paper production, continuous casting, stretching of plastic films, cooling of electronic chips and crystal growing etc. Khan et al. [3] explored analysis of heat and mass transfer in three-dimensional nanofluids flowing on a linear stretching sheet under convective wall conditions and thermal radiations. It was deduced that heat and mass transfer rates enhance with the stretching parameter. Gireesha et al. [4] investigated the influence of non-linear radiation on MHD boundary layer dynamics of a Jeffrey nanofluid past a non-linear porous stretching plate. Three-dimensional flow of fluid was considered. It was found that magnetic field diminishes the fluid velocity while it enhances temperature. Hayat et al. [5] analyzed the influence of magnetic induction on dynamics of second-grade nanofluid due to stretching sheet with convective wall conditions. The viscoelastic parameter was observed to upsurge the fluid parameter. Rafiq et al. [6] exposed the effects of non-linear thermal radiation towards boundary layer dusty fluid dynamics close to a rotating isothermally heated blunt-nosed object similar to a hemisphere. It was determined that skin friction coefficient shows an asymptotic trend while heat transfer coefficient increases significantly corresponding to large radiation parameter. Khan et al. [7] analytically studied a mixed convection hybrid nanofluid consisting of Al_2_O_3_ and Ag nanoparticles affected by induced magnetic field in order to analyze entropy generation under viscous dissipation and heat generation effects. They observed that viscous dissipation dominantly increases flow and heat transfer rate due to the no-slip surface condition. Few other attempts in this regime are cited here [8,9,10,11,12,13,14].

Technological developments and increasing demand of optical and electronic equipment required an improved cooling performance of the products which is acquired by utilizing heat transfer fluids with the improvements in their thermo physical characteristics. In order to obtain modified results, there have been plenty of endeavors by researchers to synthesize these fluids for an efficient heat transfer rate using the composition of several fluids as well as the dispersion of metallic particles of different sizes and shapes etc. Recently, an upsurge of embedding thermal resistive and conductive nano-particles, initially introduced by Choi [15], has been implemented to enhance thermal characteristics of working fluids. Liquids containing suspended nanoparticles, named as nanofluids, were experimentally guaranteed to possess their higher thermal conductivities than that of base fluids and may enable the operation of cooling systems for practical use in many fields such as in micro-electromechanical systems, pharmaceutical procedures, heat transfer, hybrid-powered engines and in field of nanotechnology. Dynamics of a magneto convective Casson nanofluid caused by Stefen blowing with bio active mixers was theoretically inspected by Puneeth et al. [16]. Awais et al. [17] theoretically exposed the rheological behavior of copper-water nanofluid peristaltic flowthrough generalized compliant walls in order to inspect influence of Hall and slip with temperature dependent viscosity. It was deduced that first and second order velocity slip parameters significantly increase flow velocity whereas rate of heat transfer is maximum in the vicinity of channel boundaries. An experimental study on the rheological characteristics of nanofluids manufactured by dispersing multi-walled carbon nanotubes in liquid paraffin was carried out by Liu et al. [18]. It was revealed that velocity components enlarge for velocity slip parameters while temperature-dependent viscosity has shown an impact of increasing temperature. In order to characterize the solar energy storage, improvement in thermal capacity of binary nitrate eutectic salt-silica nanofluid was studied by Hu et al. [19]. Relevant literature in the regime of nanofluids under several aspects can be found in [20,21,22]. Regardless of the noteworthy consequences of researchers’ endeavors, authentic applications require transaction in dissimilar characteristics of nanofluids and therefore hybrid nanofluids were synthesized by embedding special nanoparticles in base fluid. Such fluids possess improved physical and chemical properties in a homogeneous phase. Waini et al. [23] inspected MHD flow dynamics and heat transport of a hybrid nanofluid over a porous stretching/shrinking wedge. A drop of the heat transport rate was determined with the rise in radiation parameter. The temporal stability analysis was presented to evaluate the dual solutions’ stability, and it was revealed that one of the dual stables is reliable physically. Parveen et al. [24] utilized computational intelligence techniques in order to analyze heat transfer rate and pressure rise behavior in hybrid nanofluid dynamics influenced by magnetic induction effects past an endoscope. It was shown that coefficient of heat transfer accelerates toward Br and χ. Accuracy and stability of experimental data were established by employing neural network algorithm and very reliable results were obtained. Radhika et al. [25] explained the effects of magnetic field and melting heat transfer in the dynamics of dusty fluid suspended with hybrid nanoparticles. It was revealed that thermal gradient enhances for high values of magnetic parameter and Prandtl number. Reddy et al. [26] carried out theoretical analysis for heat transfer in dusty fluid dynamics suspended with hybrid nanoparticles by taking the Cattaneo-Christov heat flux model. Khan et al. [27] studied sustainability based performance evaluation of hybrid nanofluid assisted machining. Their theoretical study showed that a very small portion of nanoparticles affect the cost of each industrial product and the study was in complete accordance with the industrial applications of nanofluids. Literature in this area is shown in the references [28,29,30,31]. Moreover, the shape factor can approximately portray the difference of shape among non-spherical and spherical nanoparticles. In general, the nanoparticles possess polyhedral shapes, and their surface is made up of various planes. Thermophysical properties of nanoparticles directly depend on shape of nanoparticles. Therefore, flow and heat transport rates are examined in terms of coefficient of skin friction and Nusselt number for the nanoparticles of platelets shape with *s* = 5.7 in this analysis.

Entropy generation analysis is one of the vital factors in fluid mechanics. Performance of thermal devices directly depends upon the available amount of work which degrades by flow irreversibility and causes more disorder. Therefore, the study of dynamics behind entropy production becomes necessary in order to optimize thermal efficiencies of devices. Dogonchi et al. [32] inspected entropy generation behavior in natural convective hybrid nanofluid rheology by considering the effects of applied magnetic field past a porous cavity with wavy walls embedded in three circular cylinders. Sahoo et al. [33] carried out the analysis of entropy optimization, with dissipative heat transfer, in mixed convective MHD Casson nanofluid dynamics under the influences of Hall current and thermal radiation. Results showed that entropy generation amplifies significantly for diffusive variable, Brinkman number, and concentration ratio parameter whereas Bejan number decreases for all these parameters. In this regard, some investigations on entropy generation analysis for different flows and geometries under various physical aspects are reviewed (see articles [34,35,36]). Moreover, use of an analytical technique for the solution of the mathematical model is aimed by using homotopy analysis method (HAM). HAM, intended by Shi Jun Liao in 1992, depends on the fundamental concept of topology and differential geometry, homotopy. Being an analytical technique, HAMs are able to solve algebraic, ordinary/partial differential and differential-integral, and linear/non-linear equations in terms of series sum. It provides a broader way for selection of its arguments like initial guess, linear operator and convergence control parameter, which can be highly effective to control convergence rate of the solutions. This characteristic of HAM makes it preferable toother analytical techniques.

**Figure 1:**
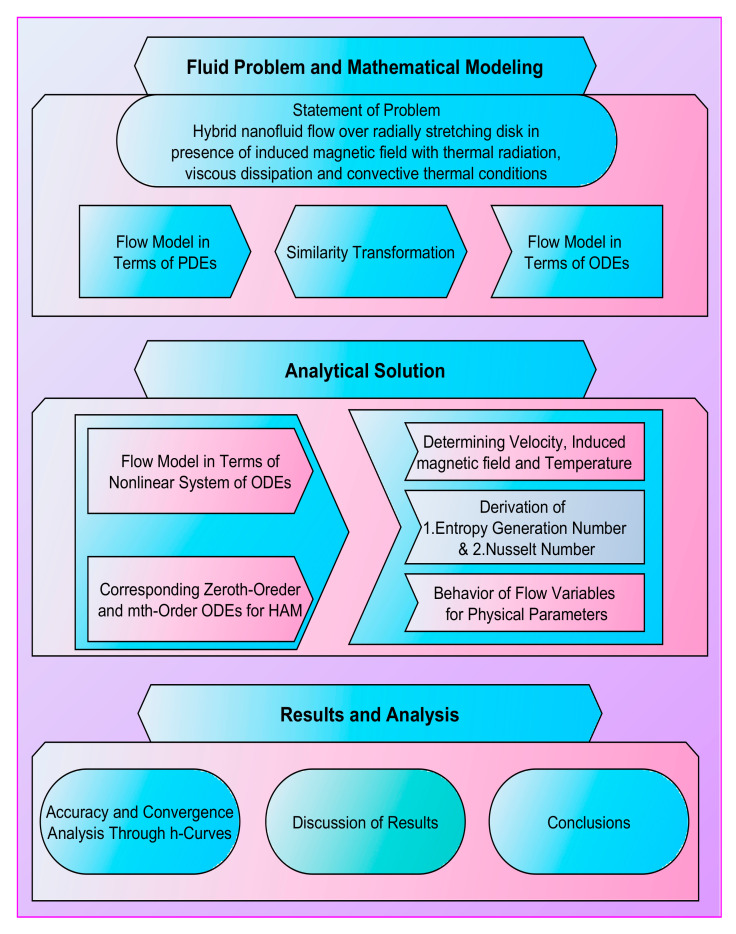
Workflow chart.

The objectives of this study are to theoretically analyze entropy generation and rate of heat transfer in steady flow of (Al_2_O_3_-Cu/H_2_O) hybrid nanofluid induced due to radially stretching disk by imposing convective-type thermal conditions. Flow is axisymmetric in which all the flow variables are independent of angular dimension. The influence of induced magnetic field is taken into account. Flow is considered in the presence of viscous dissipation and thermal radiation effects. The complete mechanism of the present study is explored in terms of a workflow diagram in Figure 1.

## 2. Modeling and Problem

**Figure 2:**
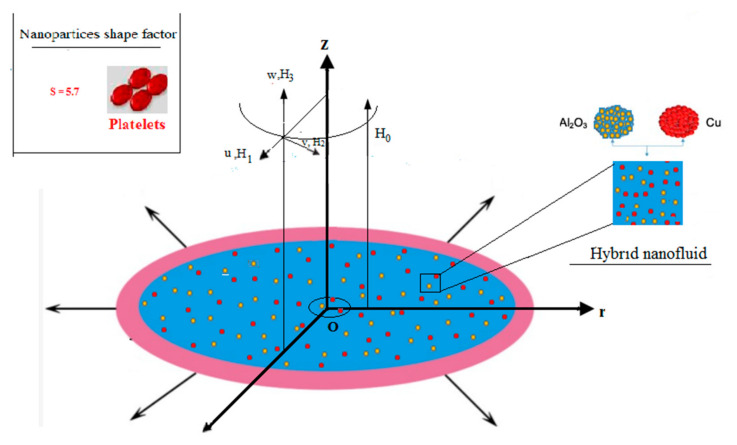
Schematic representation.

**Figure 3:**
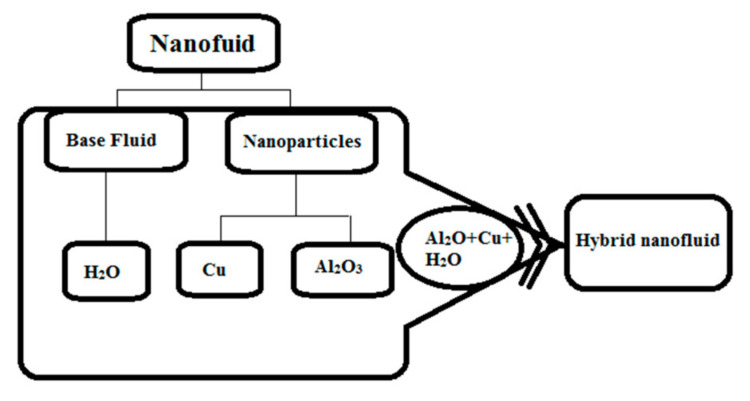
Preparation scheme of nanofluid and hybrid nanofluids.

Steady, boundary layer flow of viscous (Al_2_O_3_-Cu/H_2_O) hybrid nanofluid induced due to stretching disk in radial directions is assumed. The volume concentration of Al_2_O_3_ and Cu nanomaterials is taken to be 0.05%. The stretching velocity of the disk surface is *U_w_*(*r*) *= ar* where a represents positive constant. The surface of disk is convectively heated by fluid having temperature T and held in plane *z* = 0 while hybrid nanofluid is flowing in the region *z* > 0 as shown in Figure 2. Moreover, a scheme for manufacturing of nanofluid and hybrid nanofluid for the nanoparticles in the present study is presented in Figure 3.

The constitutive governing model along with induced magnetic field, thermal radiations and viscous dissipation effects under boundary layer approximation is:

(1) $$\frac{\;\partial u}{\partial r}+\frac{u}{r}+\frac{\partial w}{\partial z}=0,$$

(2) $$\frac{\;\partial H_{1}}{\partial r}+\frac{H_{1}}{r}+\frac{\partial H_{3}}{\partial z}=0,$$

(3) $$u\frac{\;\partial u}{\partial r}+w\frac{\partial u}{\partial z}-\frac{\hat{\mu }}{4\pi \rho_{f}}\left(H_{1}\frac{\;\partial H_{1}}{\partial r}+H_{3}\frac{\partial H_{1}}{\partial z}\right)=-\frac{\hat{\mu }}{4\pi \rho_{f}}H_{e}\frac{\;dH_{e}}{dr}+\left(\frac{\mu_{hnf}}{\rho_{hnf}}\right)\frac{\partial^{2}u}{\partial z^{2}},$$

(4) $$u\frac{\;\partial H_{1}}{\partial r}+w\frac{\partial H_{1}}{\partial z}-H_{1}\frac{\;\partial u}{\partial r}-H_{3}\frac{\partial u}{\partial z}=\mu_{e}\frac{\partial^{2}H_{1}}{\partial z^{2}},$$

(5) $$u\frac{\;\partial T}{\partial r}+w\frac{\partial T}{\partial z}=\alpha_{hnf}\frac{\partial^{2}T}{\partial z^{2}}+\left(\frac{\mu_{hnf}}{{\left(\rho c_{p}\right)}_{hnf}}\right){\left(\frac{\partial u}{\partial z}\right)}^{2}-\left(\frac{1}{{\left(\rho c_{p}\right)}_{hnf}}\right)\frac{\partial q_{r}}{\partial z},$$

Radial, axial and azimuthal components of the induced magnetic field vector are, *H*_1_, *H*_2_ and *H*_3_, respectively. Corresponding boundary conditions are:

(6) $$\begin{array}{l} u=U_{w}\left(r\right)=ar,\;-\kappa_{hnf}\frac{\partial T}{\partial z}=h\left(T_{w}-T\right),\;w=0,\;\frac{\partial H_{1}}{\partial z}=0,\\ \;H_{2}=H_{3}=0,\;at\;z=0,\\ u=w=0,\;T\to T_{\infty },\;H_{1}=H_{e}\left(r\right)=H_{0}r,\;as\;z\to \infty . \end{array}$$

In Equation (5), expression for *q_r_* by using Roseland approximation and Taylor series expansion of *T*^4^ about *T*_∞_ can be expressed as:

(7) $$q_{r}=-\frac{4\sigma }{3k}\frac{\partial T^{4}}{\partial z}=-\frac{16\sigma T_{\infty }{}^{3}}{3k}\frac{\partial T}{\partial z},$$

Now, using similarity transformation:

(8) $$\begin{array}{l} u\left(r,z\right)=arf^{\prime }\left(\eta \right),\;w(r,z)=-2\sqrt{a\upsilon_{f}}f\left(\eta \right),\;\eta =\sqrt{\frac{a}{\upsilon_{f}}}z,\\ H_{1}=H_{0}rg^{\prime }\left(\eta \right),{\;H}_{3}=-2\sqrt{a\upsilon_{f}}g\left(\eta \right),\;\theta \left(\eta \right)=\frac{T-T_{\infty }}{T_{w}-T_{\infty }}. \end{array}$$

For the above transformations, Equations (1) and (2) are identically satisfied while Equations (3)–(5) within boundary conditions (6) and Equation (7) gives:

(9) $$f^{\prime \prime \prime }-\Phi_{1}\Phi_{2}\{{f^{\prime }}^{2}-2ff^{\prime \prime }-\beta ({g^{\prime }}^{2}-2gg^{\prime \prime }-1)\}=0,$$

(10) $$\lambda g^{\prime \prime \prime }+2fg^{\prime \prime }-2f^{\prime \prime }g=0,$$

(11) $$(\Phi_{4}+\frac{4}{3}R)\theta^{\prime \prime }+2\Phi_{3}Pr.f\theta^{\prime }+\frac{Pr\cdot Ec}{\Phi_{1}}{f^{\prime \prime }}^{2}=0$$.

The transformed boundary conditions are:

(12) $$\begin{array}{l} f\left(\eta \right)=g\left(\eta \right)=0,\;f^{\prime }\left(\eta \right)=1,\;g^{\prime \prime }\left(\eta \right)=0,\;\theta^{\prime }\left(\eta \right)=-\frac{Bi}{\Phi_{4}}\left(1-\theta \left(0\right)\right),\;at\;\eta =0,\\ f^{\prime }\left(\eta \right)\to 0,\;g^{\prime }\left(\eta \right)\to 1,\;\theta \left(\eta \right)\to 0,\;as\;\eta \to \infty . \end{array}$$

where prime represents differentiation with
respect to 

Moreover, expressions for thermophysical properties are:

(13) $$\begin{array}{c} \Phi_{1}={\left(1-\varphi_{1}\right)}^{2.5}{\left(1-\varphi_{2}\right)}^{2.5},\;\Phi_{2}=\left(1-\varphi_{2}\right)\left[\left(1-\varphi_{1}\right)+\varphi_{1}\left(\frac{\rho_{s_{1}}}{\rho_{f}}\right)\right]+\varphi_{2}\left(\frac{\rho_{s_{2}}}{\rho_{f}}\right),\\ \Phi_{3}=\left(1-\varphi_{2}\right)\left[\left(1-\varphi_{1}\right)+\varphi_{1}\left(\frac{{\left(\rho c_{p}\right)}_{s_{1}}}{{\left(\rho c_{p}\right)}_{f}}\right)\right]+\varphi_{2}\frac{{\left(\rho c_{p}\right)}_{s_{2}}}{{\left(\rho c_{p}\right)}_{f}},\\ \Phi_{4}=\frac{\kappa_{s_{2}}+\left(s-1\right)\kappa_{bf}-\left(s-1\right)\varphi_{2}\left(\kappa_{bf}-\kappa_{s_{2}}\right)}{\kappa_{s_{2}}+\left(s-1\right)\kappa_{bf}+\varphi_{2}\left(\kappa_{bf}-\kappa_{s_{2}}\right)}\frac{\kappa_{s_{1}}+\left(s-1\right)\kappa_{f}-\left(s-1\right)\varphi_{1}\left(\kappa_{f}-\kappa_{s_{1}}\right)}{\kappa_{s_{1}}+\left(s-1\right)\kappa_{f}+\varphi_{1}\left(\kappa_{f}-\kappa_{s_{1}}\right)}, \end{array}$$

Moreover, all the thermophysical characteristics of nanoparticles and base fluid are mentioned in Table 1 while expressions for dimensionless parameters are:

(14) $$\begin{array}{l} Pr=\frac{{\left(\mu c_{p}\right)}_{f}}{\kappa_{f}},\;\lambda =\frac{\mu_{e}}{\upsilon_{f}},\;\beta =\frac{\hat{\mu }}{4\pi \rho_{f}}{\left(\frac{H_{0}}{a}\right)}^{2},\\ Ec=\frac{U_{w}{}^{2}}{{\left(c_{p}\right)}_{f}\left(T_{w}-T_{\infty }\right)},\;R=\frac{4\sigma T_{\infty }{}^{3}}{k\kappa_{f}},\;Bi=\frac{h}{\kappa_{f}}\sqrt{\frac{\upsilon_{f}}{a}}, \end{array}$$

Expressions for coefficient of skin friction *C_f_* and local Nusselt number *Nu* are:

(15) $$\;C_{f}=\frac{\tau_{w}}{\rho_{f}{\left(U_{w}\right)}^{2}}\;,Nu=-\frac{rq_{w}}{\kappa_{f}\left(T_{w}-T_{\infty }\right)}.$$

In the above expressions, *τ_w_* and *q_w_* symbolize shear stress and heat flux for wall, respectively. The dimensionless form by substituting Equation (8) is:

(16) $${Re}_{r}{}^{\frac{1}{2}}C_{f}=\frac{-1}{\Phi_{1}}f^{\prime \prime }\left(0\right),\;{Re}_{r}{}^{\frac{-1}{2}}Nu=-\left(\Phi_{4}+\frac{4}{3}R\right)\theta^{\prime }\left(0\right),$$

where, ${Re}_{r}=\frac{U_{w}r}{\upsilon_{f}}$ indicates local Reynolds number.

By adopting the second law of thermodynamics, volumetric entropy generation rate in existence of radiative and dissipative factors can be expressed as:

(17) $${{\dot{S}}^{\prime \prime \prime }}_{Gen}=\left(\Phi_{4}+\frac{4}{3}R\right)\frac{\kappa_{f}}{T_{\infty }^{2}}{\left(\frac{\partial T}{\partial y}\right)}^{2}+\frac{\mu_{f}}{\Phi_{1}T_{\infty }}{\left(\frac{\partial u}{\partial y}\right)}^{2}+\frac{\mu_{f}}{\Phi_{1}T_{\infty }}\left(\frac{\hat{\mu }}{4\pi \rho_{f}}\right){\left(\frac{\partial H_{1}}{\partial y}\right)}^{2}.\;$$

The expression for characteristic entropy generation rate is:

(18) $${{\dot{S}}^{\prime \prime \prime }}_{0}=\frac{\kappa_{f}}{T_{\infty }^{2}L^{2}}{\left(T_{w}-T_{\infty }\right)}^{2}.$$

Utilizing similarity transformation with Equation (18) in Equation (17), we have:

(19) $$N_{S}=\frac{{{\dot{S}}^{\prime \prime \prime }}_{Gen}}{{{\dot{S}}^{\prime \prime \prime }}_{0}}=\left(\Phi_{4}+\frac{4}{3}R\right){Re}_{L}.{\theta^{\prime }}^{2}+\frac{\varepsilon .Pr.Ec.{Re}_{L}}{\Phi_{1}}{f^{\prime \prime }}^{2}+\frac{\varepsilon .Pr.Ec.{Re}_{L}.\beta }{\Phi_{1}}{g^{\prime \prime }}^{2}.$$

where, *Ns* is non-dimensional form of entropy generation number, ${Re}_{L}=\frac{aL^{2}}{\upsilon_{f}}$ and $\varepsilon =\frac{T_{\infty }}{T_{w}-T_{\infty }}$ demonstrate local Reynolds number and temperature ratio parameter, respectively.

## 3. Homotopy Analysis Method and Convergence of Solutions

The dimensionless governing model mentioned in Equations (9)–(11) and subjected boundary conditions of Equation (12) for boundary layer flow of hybrid nanofluid over the stretching disk is analytically solved by employing the homotopy analysis method [7,37,38,39]. For flow variables, initial guesses are:

(20) $$f_{0}\left(\eta \right)=1-\exp\left(-\eta \right),g_{0}\left(\eta \right)=\eta ,\;\theta_{0}\left(\eta \right)=\frac{Bi}{1+Bi}\exp\left(-\eta \right).$$

Expressions for linear operators are:

(21) $$T_{1}\left(\eta \right)=f^{\prime \prime \prime }\left(\eta \right)-f^{\prime }\left(\eta \right),{\;T}_{2}\left(\eta \right)=g^{\prime \prime \prime }\left(\eta \right)+g^{\prime \prime }\left(\eta \right){,\;T}_{3}\left(\eta \right)=\theta^{\prime \prime }\left(\eta \right).$$

and

(22) $$T_{1}\left(A_{1}+A_{2}e^{-\eta }-A_{3}e^{-\eta }\right)=T_{2}\left(A_{4}+A_{5}\eta +A_{6}e^{-\eta }\right)=T_{3}\left(A_{7}+A_{8}\eta \right)=0.$$

where, *A_1_–A_8_* represents constants in general solutions.

### 3.1. Convergence-Control Parameters

Suppose an *h* is auxiliary parameter in the frame of HAM which directly affects the convergence of solutions. Let $\xi \in \left[0,1\right]$ be an embedding parameter, then the problem for zeroth order deformation is constructed as:

(23) $$\left(1-\xi \right)T_{1}\left[f\left(\eta ,\xi \right)-f_{0}\left(\eta \right)\right]=\xi hR_{1}\left[f\left(\eta ,\xi \right),g\left(\eta ,\xi \right),\theta \left(\eta ,\xi \right)\right],$$

(24) $$\left(1-\xi \right)T_{2}\left[g\left(\eta ,\xi \right)-g_{0}\left(\eta \right)\right]=\xi hR_{2}\left[f\left(\eta ,\xi \right),g\left(\eta ,\xi \right),\theta \left(\eta ,\xi \right)\right],$$

(25) $$\left(1-\xi \right)T_{3}\left[\theta \left(\eta ,\xi \right)-\theta_{0}\left(\eta \right)\right]=\xi hR_{3}\left[f\left(\eta ,\xi \right),g\left(\eta ,\xi \right),\theta \left(\eta ,\xi \right)\right].$$

Furthermore,

(26) $$\begin{array}{l} \mathrm{at}\;\eta =\eta_{1}=0,\;f\left(\eta_{1},\xi \right)=g\left(\eta_{1},\xi \right)=0,\;f^{\prime }\left(\eta_{1},\xi \right)=1,\;g^{\prime \prime }\left(\eta_{1},\xi \right)=0,\;\theta^{\prime }\left(\eta_{1},\xi \right)=-\frac{Bi}{A_{4}}\left(1-\theta \left(\eta_{1},\xi \right)\right).\\ \mathrm{At}\;\eta =\eta_{2}\to \infty ,f^{\prime }\left(\eta_{2},\xi \right)\to 0,g^{\prime }\left(\eta_{2},\xi \right)\to 1,\;\theta \left(\eta_{2},\xi \right)\to 0 \end{array}$$.

Using the aforementioned quantities, the *m*th order solution series is constructed as:

(27) $$T_{1}\left[f_{m}\left(\eta ,\xi \right)-\varsigma_{m}f_{m-1}\left(\eta ,\xi \right)\right]=hS_{m}^{1}\left[\left(\eta ,\xi \right)\right],$$

(28) $$T_{2}\left[g_{m}\left(\eta ,\xi \right)-\varsigma_{m}g_{m-1}\left(\eta ,\xi \right)\right]=hS_{m}^{2}\left[\left(\eta ,\xi \right)\right],$$

(29) $$T_{3}\left[\theta_{m}\left(\eta ,\xi \right)-\varsigma_{m}\theta_{m-1}\left(\eta ,\xi \right)\right]=hS_{m}^{3}\left[\left(\eta ,\xi \right)\right].$$

In the above equations,

$$\varsigma_{m}=\left.\begin{array}{l} 0m\leq 1\\ 1m\;>1 \end{array}\right]$$

Subjected boundary conditions are:

(30) $$\begin{array}{l} \mathrm{At}\;\eta =\eta_{1}=0,\;f_{m}\left(\eta_{1},\xi \right)=g_{m}\left(\eta_{1},\xi \right)=0,\;{f^{\prime }}_{m}\left(\eta_{1},\xi \right)=0,\;{g^{\prime \prime }}_{m}\left(\eta_{1},\xi \right)=0,\;\\ \;\;\;\;\;\theta_{m}\left(\eta_{1},\xi \right)=0,\\ \mathrm{At}\;\eta =\eta_{2}\to \infty ,{f^{\prime }}_{m}\left(\eta_{2}\right)\to 0,{g^{\prime }}_{m}\left(\eta_{2}\right)\to 0,\;\theta_{m}\left(\eta_{2}\right)\to 0 \end{array}$$.

Then, we can write:

(31) $$\begin{array}{l} f_{m}\left(\eta ,\xi \right)=F_{m}\left(\eta ,\xi \right)+A_{1}+A_{2}e^{-\eta }-A_{3}e^{-\eta },\\ g_{m}\left(\eta ,\xi \right)=G_{m}\left(\eta ,\xi \right)+A_{4}+A_{5}\eta +A_{6}e^{-\eta },\\ \theta_{m}\left(\eta ,\xi \right)=\Theta_{m}\left(\eta ,\xi \right)+A_{7}+A_{8}\eta . \end{array}$$

### 3.2. Convergence of Solutions

**Figure 4:**
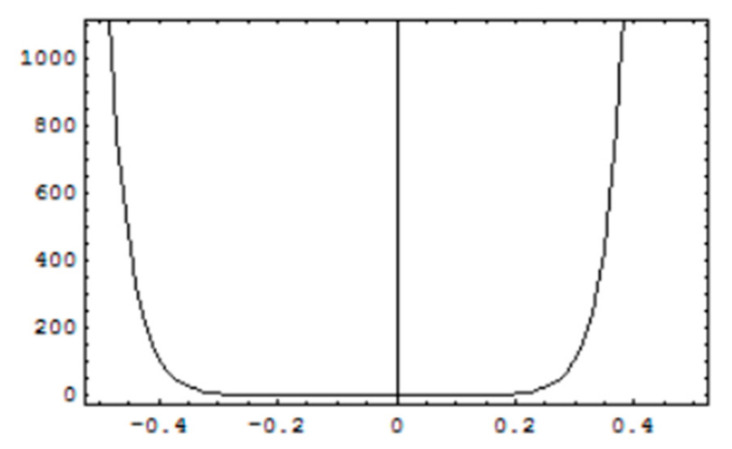
*h*-curves for solution of $f^{\prime }\left(0\right)$.

**Figure 5:**
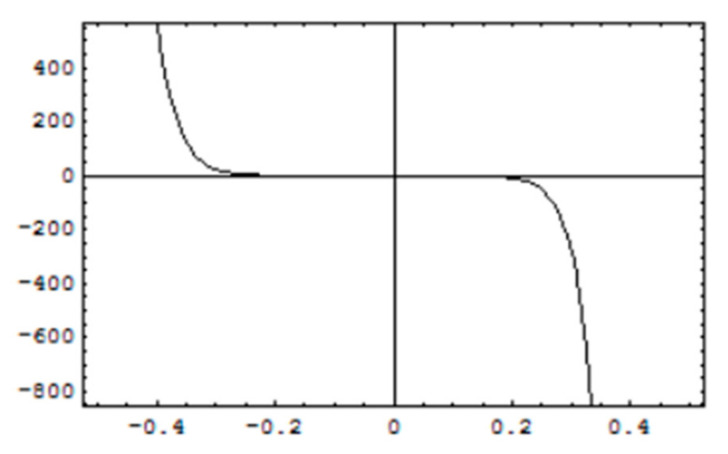
*h*-curves for solution of $g^{\prime }\left(0\right)$.

**Figure 6:**
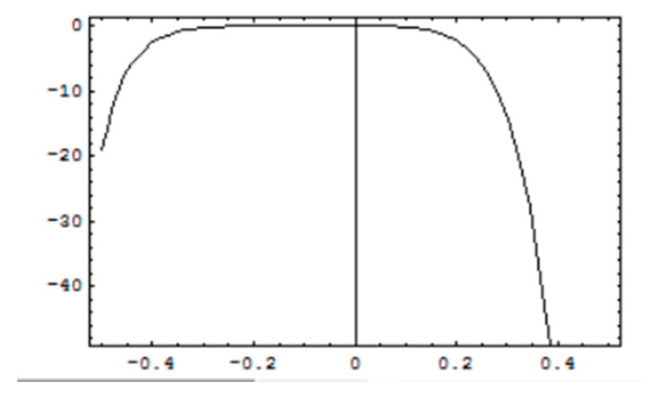
*h*-curves for the solution of $\theta \left(0\right)$.

For stability and convergence of analytical solutions, h-curves for, $f^{\prime }\left(0\right)$ $g^{\prime }\left(0\right)$ and $\theta \left(0\right)$ are prepared upto 20th order solutions by plotting the interval of convergence and are shown in Figure 4, Figure 5 and Figure 6. Convergence control parameter *h* is able to control and adjust convergence region of HAM solutions. It is observed from convergence analysis that valid convergence region is *R_h_* = [−0.4, 0.2]. For $f^{\prime }\left(0\right)$, *R_h_* = [−0.35, 0.2] for $g^{\prime }\left(0\right)$, and *R_h_* = [−0.35, 0.15] for $\theta \left(0\right)$. Moreover, testing various values of *h* from corresponding regions, the error is minimum at *h* = −0.1 which guarantees that calculated solutions are very accurate with a negligible error and become more accurate for higher-order solutions.

## 4. Discussion of Results

**Figure 7:**
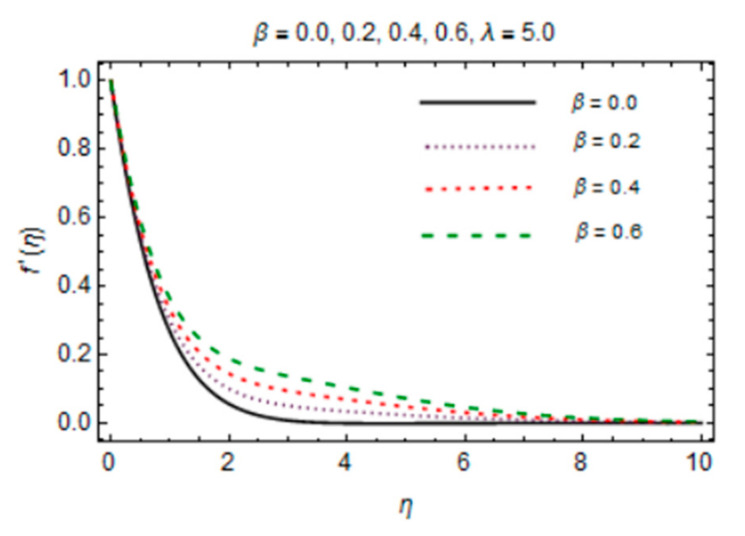
*β* verses velocity profile.

**Figure 8:**
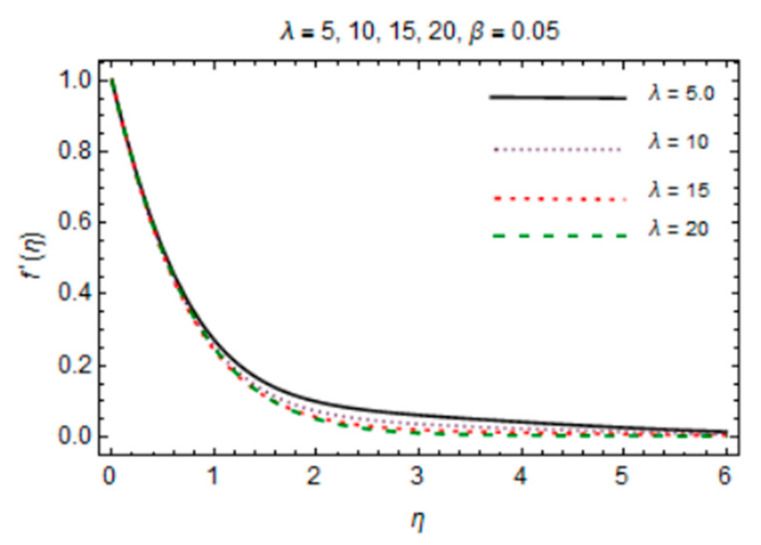
*λ* verses velocity profile.

Analytical solutions for the Al_2_O_3_-Cu/water hybrid nanofluid flow obtained by HAM are discussed in this section. Figure 7 and Figure 8 plot the variation in magnetic parameter *β* and the reciprocal of magnetic Prandtl number *λ* for the velocity profile. The graph indicates that rise in values of *β* enhances $f^{\prime }\left(\eta \right)$ due to more dominant induction effects than magnetic diffusion and the flow rate increases. A drop in $f^{\prime }\left(\eta \right)$ is noticed for *λ* because magnetic diffusivity rises with rise in *λ*. This effect causes enhancement of frictional force and the velocity boundary layer thickness reduces.

**Figure 9:**
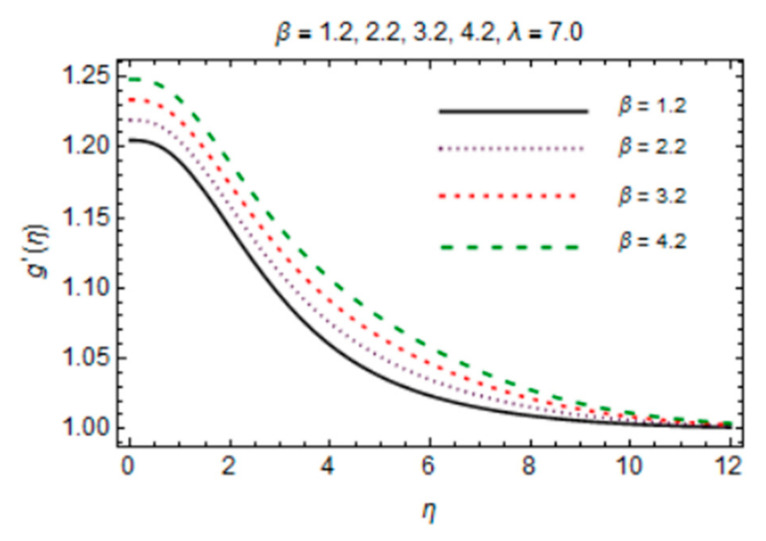
*β* verses induced magnetic field profile.

**Figure 10:**
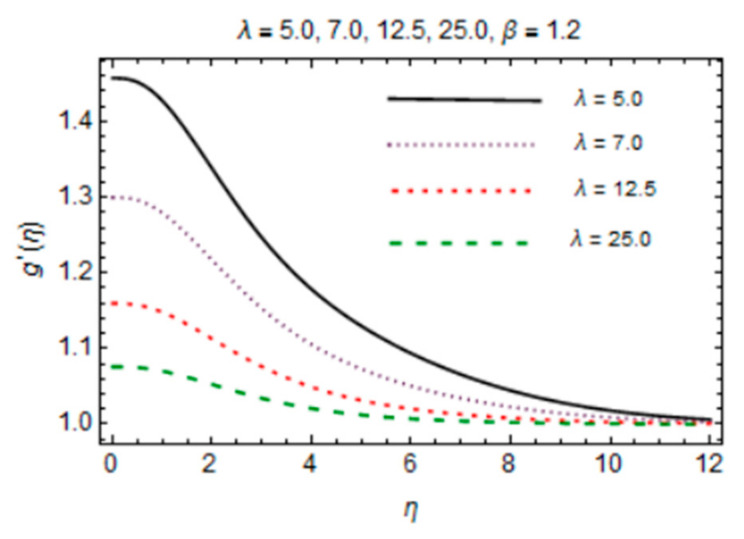
*λ* verses induced magnetic field profile.

The behavior of induced magnetic field profile against parameters *β* and *λ* is explored in Figure 9 and Figure 10, respectively. Graphs demonstrate that with augmentation in *β*, magnetic effects become strong as due to fast advection process, therefore increasing flow rate amplifies magnetic induction profile. Consecutively, $g^{\prime }\left(\eta \right)$ is a decreasing function of *λ* which is mainly due to enhancing magnetic diffusivity with high values of *λ*. It is noteworthy that the influences of parameters on $f^{\prime }\left(\eta \right)$ and $g^{\prime }\left(\eta \right)$ are more prominent at the interface and very little variation is shown near stretching disk due to the no slip boundary condition.

**Figure 11:**
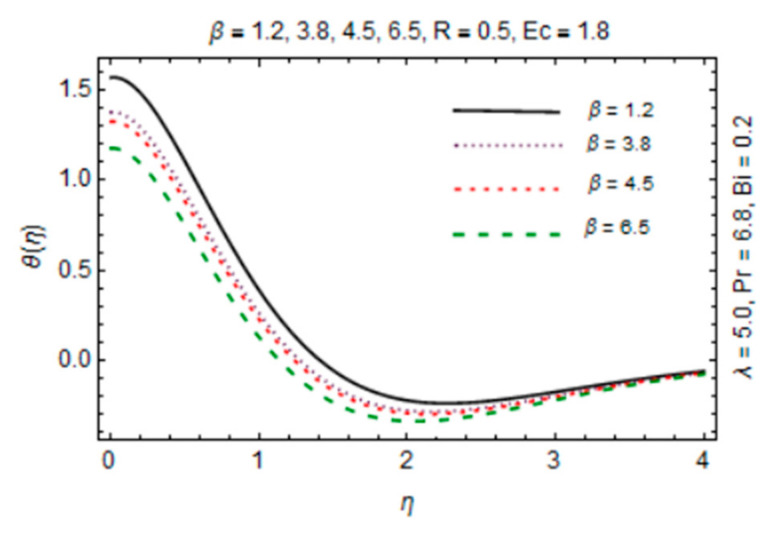
*β* verses temperature profile.

**Figure 12:**
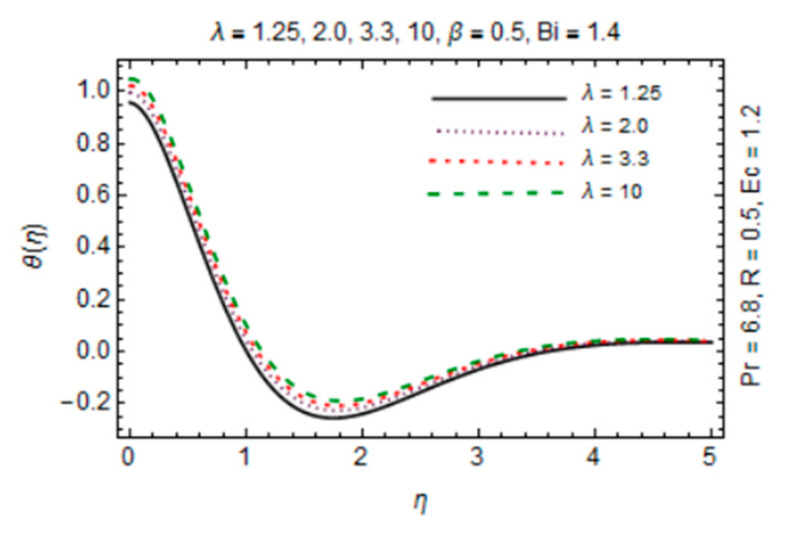
*λ* verses temperature profile.

**Figure 13:**
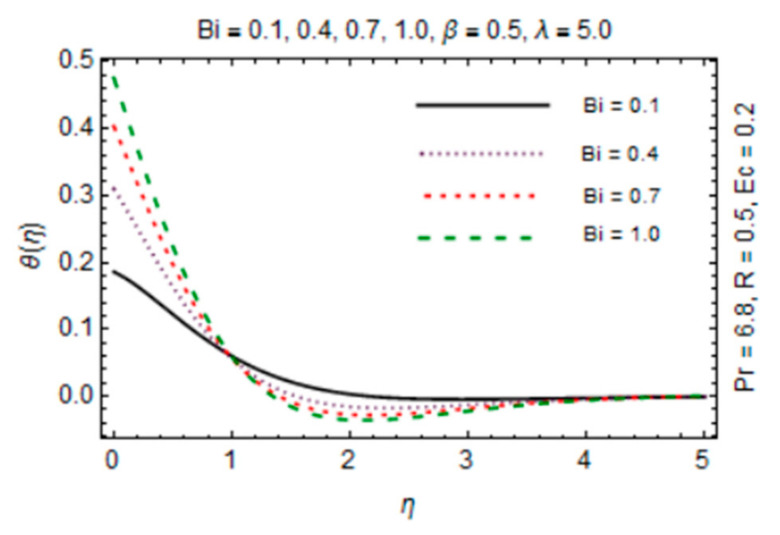
*Bi* verses temperature profile.

**Figure 14:**
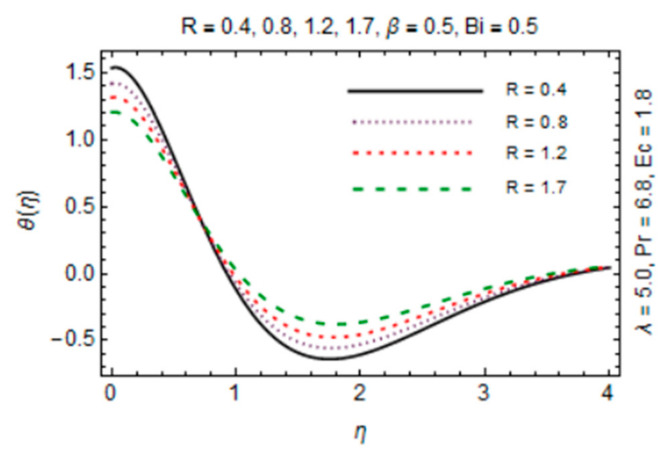
*R* verses temperature profile.

Figure 11, Figure 12, Figure 13 and Figure 14 are plotted in order to express the variation in temperature against magnetic parameter, reciprocal of magnetic Prandtl number, radiation parameter and Biot number. Results reveal that *θ*(*η*) decreases with increment in *β* which is caused by increasing heat transfer rate near the disk in existence of induced magnetic field due to adding the flow mechanism as explored in Figure 11. The impact of radiation parameter in Figure 12 depicts the dual behavior of the *θ*(*η*) profile which is depressed close to the disk and elevated away from it showing dominant effects at free stream. Since the disk is convectively heated and these effects trim down away from its surface, temperature enhances at the disk surface due to rising intensity of convective heating in Figure 13.

An opposite trend is observed in the free stream region due to the fact that additional heat released to the coolant at the surface. The impact of *λ* on temperature of hybrid nanofluid is exposed in Figure 14 which signifies that temperature rises against *λ*. The reason is high diffusivity and a small flow rate due to low magnetic induction for large magnitude of *λ*. Moreover, variation in *θ*(*η*) directly associated with values of parameters *Bi*, *R* and *Ec* which are taken to be small with *Pr* = 6.8 in this study.

**Figure 15:**
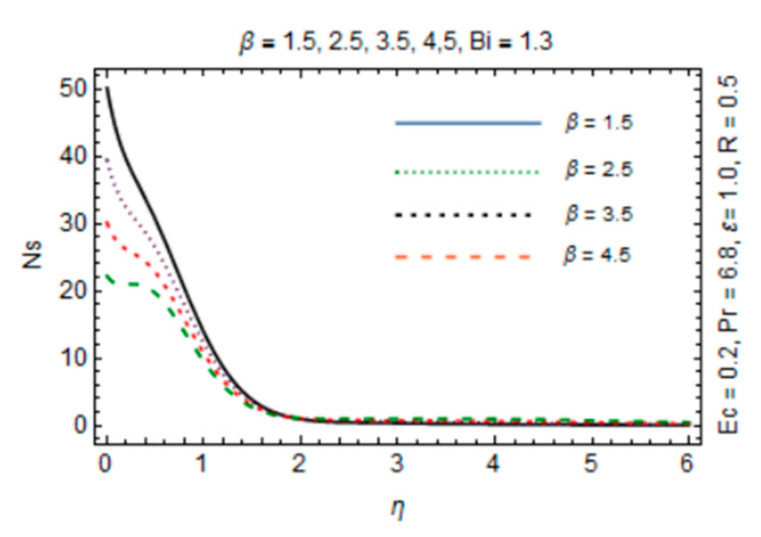
*β* verses entropy generation number.

**Figure 16:**
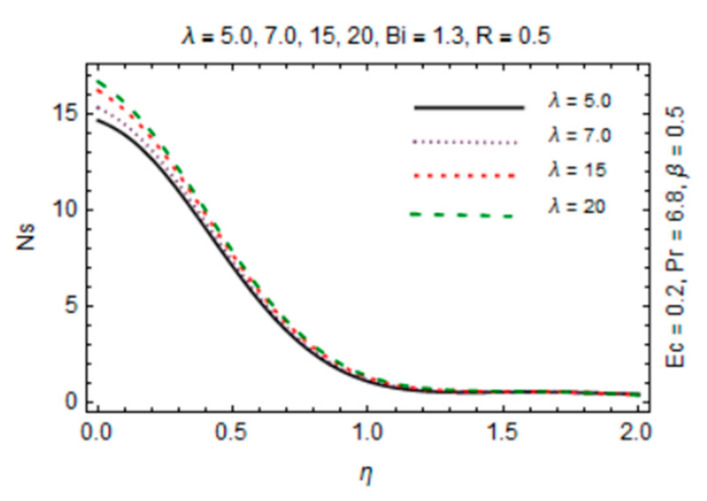
*λ* verses entropy generation number.

**Figure 17:**
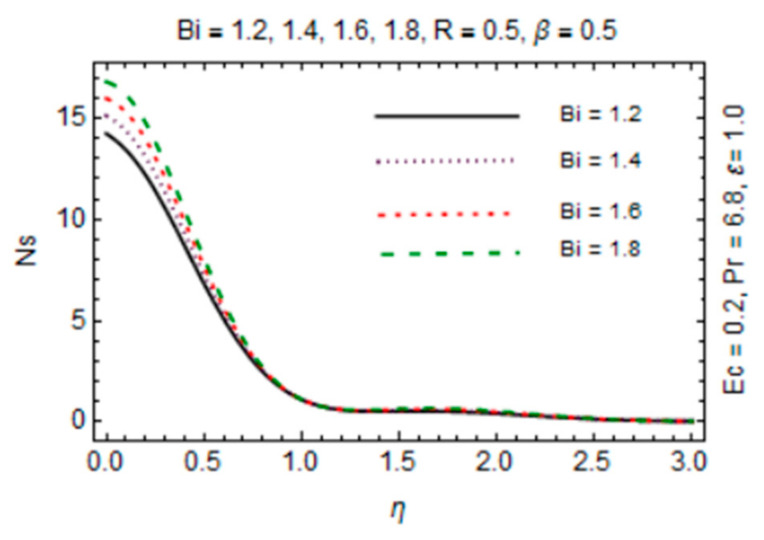
*Bi* verses entropy generation number.

**Figure 18:**
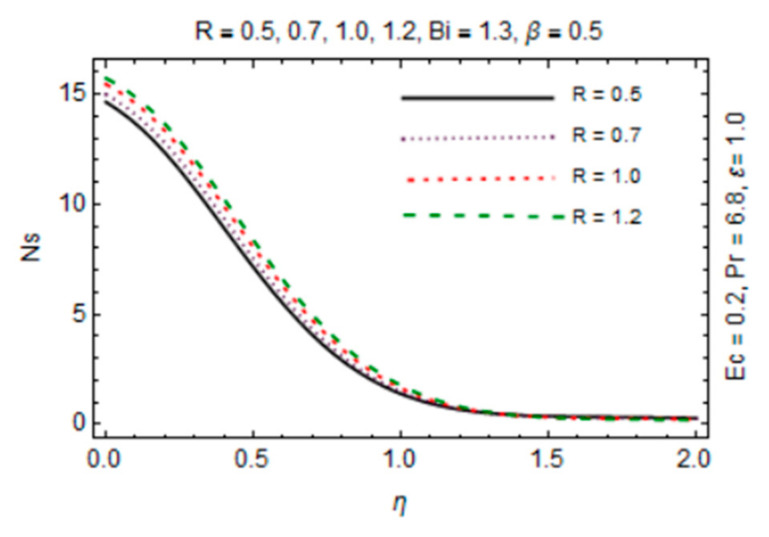
*R* verses entropy generation number.

The most persuasive part of this section is entropy generation analysis. The variation in entropy generation number Ns against emerging parameters is pointed out graphically in Figure 15, Figure 16, Figure 17 and Figure 18. Impact of magnetic parameter in Figure 15 demonstrates that rise in *β* decreases entropy production as strong effects of magnetic field close to the disk reduce the frictional effects and rise in heat transfer rate. It is of true significance physically since the rate of fluid flow enhances instantly and heat transfer rate increases. The number slightly increases in the free stream region due to decreasing fluid flow. Figure 16 expresses an enhancement in Ns as values of reciprocal of magnetic Prandtl number rises. This is because of the fact that as *λ* enhances, reduction in viscosity occurs while magnetic diffusivity accelerates, which produce disorder in the system. In Figure 17, entropy generation number is plotted against *Bi* which illustrates that for somewhat large values of parameter *Bi*, Ns have high magnitude due to strong influences of thermal convection and maximum radial gradient. Additionally, thermal radiation effects on Ns in Figure 18 displays it as an increasing function of R due to increasing emitting radiations which boosts frictional irreversibility that encourage entropy generation. Furthermore, variations in entropy generation number are drawn for small values of radiation parameter *R*, Eckert number Ec and Biot number *Bi* which are directly related to entropy production. Also, extensive behavior for Ns against parameters is observed at the interface due to large velocity and temperature gradients caused by no slip at the surface and convective wall conditions but it is rarely affected by these parameters away from it.

Moreover, empirical formulas for the thermophysical properties of nanofluid and hybrid nanofluid are displayed in Table 2. 

**Figure 19:**
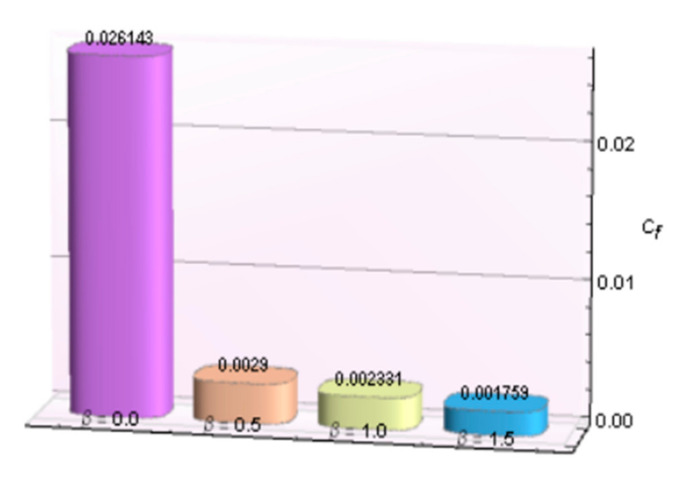
*β* verses *C_f_*.

**Figure 20:**
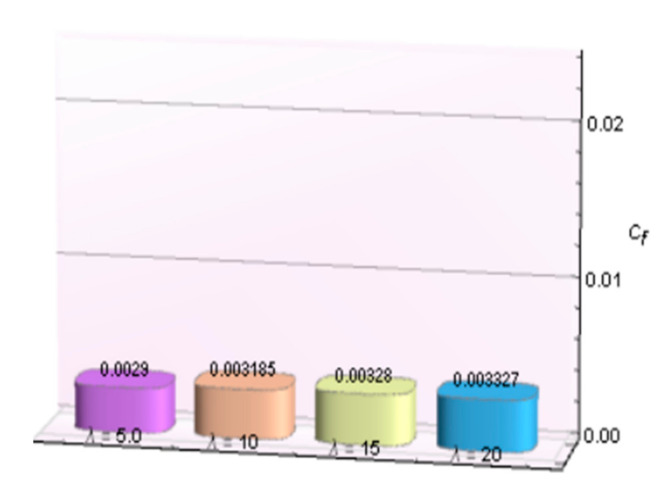
λ verses Cf.

**Figure 21:**
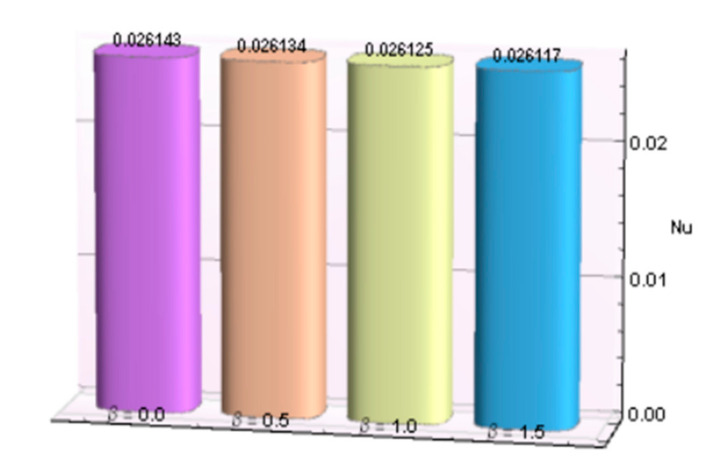
*β* verses *Nu*.

**Figure 22:**
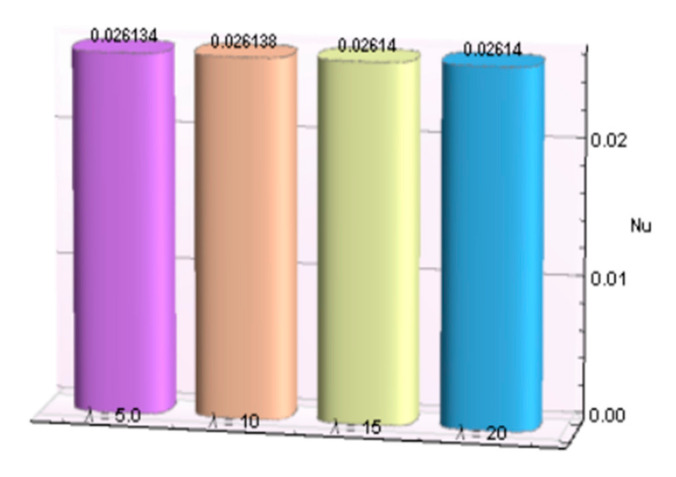
λ verses *Nu*.

**Figure 23:**
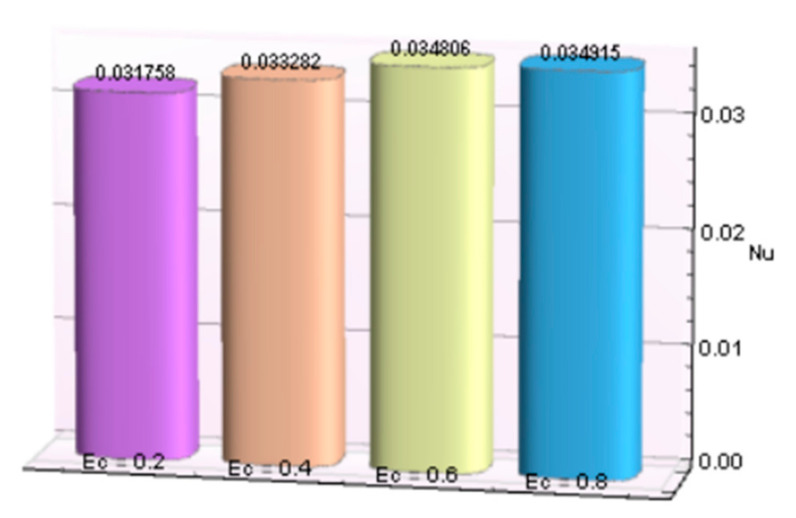
*Ec* verses *Nu*.

**Figure 24:**
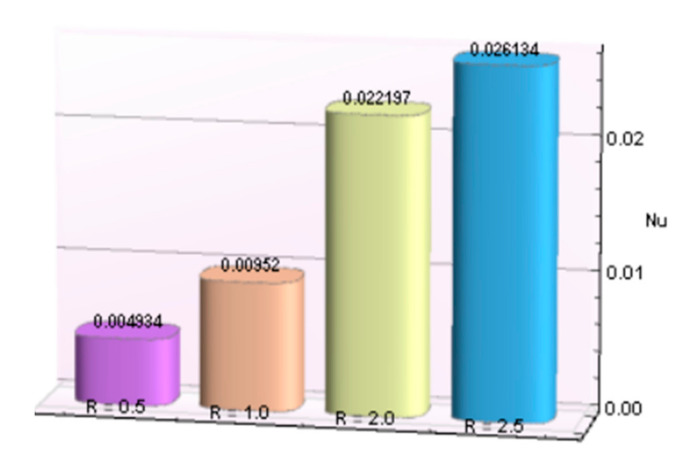
*R* verses *Nu*.

**Figure 25:**
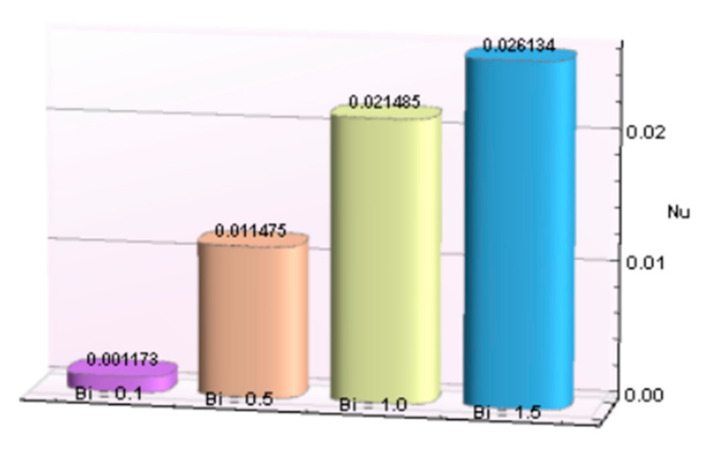
*Bi* verses *Nu*.

The impacts of involving parameters on skin friction coefficient and heat transfer rate through Table 3 and Table 4 as well as bar graphs in Figure 19, Figure 20, Figure 21, Figure 22, Figure 23, Figure 24 and Figure 25 are explained in this section. Observations conclude that the surface velocity gradient is depressed as values of magnetic parameter *β* rises but enhances with elevation in λ. Moreover, an expansion in Nusselt number is noticed for rising values of the reciprocal of magnetic Prandtl number, Biot number, radiation parameter, and Eckert number whereas it is a decreasing function of magnetic parameter.

## 5. Concluding Remarks

A theoretical discussion of MHD viscous flow of Al_2_O_3_-Cu/H_2_O hybrid nanofluid due to stretching of the disk is carried out. Some noteworthy influences of important emerging parameters on flow profiles and entropy generation are as follows:

1. $f^{\prime }\left(\eta \right)$ and $g^{\prime }\left(\eta \right)$ profiles are increasing functions of magnetic parameter while an opposite behavior is seen for enhancing values of λ.
2. Increment in *β* results in an enhancement in temperature, whereas it reduces against λ.
3. An increasing behavior for the temperature of the fluid is observed against rising values of parameters *Bi* and *R* at the surface. An opposite trend is depicted in the ambient region with a point of inflection in the field.
4. Entropy generation number enhances for enhancement in values of parameters *Bi*, *R* and λ but diminishes against *β*.
5. Flow profiles and entropy generation number are large near the surface of the disk and then decrease asymptotically far away from it. Also, these are more sensitive to fluctuate at the interface for several involved parameters.
6. At the interface, fluid temperature is significantly different from ambient temperature. Thus, convection at the wall corresponding to high values of convective heat coefficient leads to increased rate of heat transfer at the interface.
7. Values of skin friction and heat transfer coefficient can be optimized by choosing suitable values of involved parameters regarding different physical problems.

## Figures and Tables

**Table 1:** Experimental values of various thermophysical properties for base fluid and nanoparticles [27].

** Properties\Constituents **	** H_2_O **	** Al_2_O_3_**	** Cu **
Density, *ρ* (Kg/m^3^)	997	3970	8933
Specific heat, *C_p_* (J/kg K)	4179	765	385
Thermal conductivity, *κ* (W/m K)	0.613	40	401

**Table 2:** Formulas for thermophysical properties of nanofluid and hybrid nanofluid [40].

**Properties**	**Nanofluid**	**Hybrid Nanofluid**
Density	$\rho_{nf}=\rho_{f}\left[\left(1-\varphi \right)+\varphi \left(\frac{\rho_{s}}{\rho_{f}}\right)\right]$	$\rho_{hnf}=\rho_{f}\left(1-\varphi_{2}\right)\left[\left(1-\varphi_{1}\right)+\varphi_{1}\left(\frac{\rho_{s_{1}}}{\rho_{f}}\right)\right]+\varphi_{2}\rho_{s_{2}}$
Heat Capacity	${\left(\rho c_{p}\right)}_{nf}={\left(\rho c_{p}\right)}_{f}\left[\left(1-\varphi \right)+\varphi \left(\frac{{\left(\rho c_{p}\right)}_{s}}{{\left(\rho c_{p}\right)}_{f}}\right)\right]$	${\left(\rho c_{p}\right)}_{hnf}={\left(\rho c_{p}\right)}_{f}\left(1-\varphi_{2}\right)\left[\left(1-\varphi_{1}\right)+\varphi_{1}\left(\frac{{\left(\rho c_{p}\right)}_{s_{1}}}{{\left(\rho c_{p}\right)}_{f}}\right)\right]+\varphi_{2}{\left(\rho c_{p}\right)}_{s_{2}}$
Viscosity	$\mu_{nf}=\frac{\mu_{f}}{{\left(1-\varphi \right)}^{2.5}}$	$\mu_{hnf}=\frac{\mu_{f}}{{\left(1-\varphi_{1}\right)}^{2.5}{\left(1-\varphi_{2}\right)}^{2.5}}$
Thermal Conductivity	$\frac{\kappa_{nf}}{\kappa_{f}}=\frac{\kappa_{s}+\left(s-1\right)\kappa_{f}-\left(s-1\right)\varphi \left(\kappa_{f}-\kappa_{s}\right)}{\kappa_{s}+\left(s-1\right)\kappa_{f}+\varphi \left(\kappa_{f}-\kappa_{s}\right)}$	$\begin{array}{l} \frac{\kappa_{hnf}}{\kappa_{bf}}=\frac{\kappa_{s_{2}}+\left(s-1\right)\kappa_{bf}-\left(s-1\right)\varphi_{2}\left(\kappa_{bf}-\kappa_{s_{2}}\right)}{\kappa_{s_{2}}+\left(s-1\right)\kappa_{bf}+\varphi_{2}\left(\kappa_{bf}-\kappa_{s_{2}}\right)},\\ where\frac{\kappa_{bf}}{\kappa_{f}}=\frac{\kappa_{s_{1}}+\left(s-1\right)\kappa_{f}-\left(s-1\right)\varphi_{1}\left(\kappa_{f}-\kappa_{s_{1}}\right)}{\kappa_{s_{1}}+\left(s-1\right)\kappa_{f}+\varphi_{1}\left(\kappa_{f}-\kappa_{s_{1}}\right)} \end{array}$

**Table 3:** Behavior of skin friction coefficient for Al_2_O_3_-Cu/H_2_Oagainst several parameters.

*β*	*λ*	*−C_f_*
0.0	5.0	0.003467
0.5		0.002900
1.0		0.002331
1.5		0.001759
0.5	5.0	0.002900
	10	0.003185
	15	0.003280
	20	0.003327

**Table 4:** Behavior of Nusselt number for Al_2_O_3_-Cu/H_2_O against several parameters.

β	λ	*Bi*	*R*	*Ec*	−*N_u_*
0.0	5.0	1.5	2.5	0.2	0.026143
0.5					0.026134
1.0					0.026125
1.5					0.026117
0.5	5.0				0.026134
	10				0.026138
	15				0.026140
	20				0.026140
	5.0	0.1			0.001173
		0.5			0.011475
		1.0			0.021485
		1.5			0.026134
		1.5	0.5		0.004934
			1.0		0.00952
			2.0		0.022197
			2.5		0.026134
			2.5	0.2	0.031758
				0.4	0.033282
				0.6	0.034806
				0.8	0.034915

## Data Availability

The data presented in this study is available in article.

## Nomenclature

	**Nomenclature**		**Greek Symbols**
*a*	Constant	β	Magnetic parameter
*Bi*	Biot number	η	Dimensionless similarity variable
*c_f_*	Skin friction coefficient	φ	Nanoparticles volume fraction
*Ec*	Eckert number	κ	Thermal conductivity
*H*_0_	Uniform magnetic field	ρ	Density
*h*	Convective heat transfer coefficient	$\rho c_{p}$	Heat capacity
*k*	Mean absorption coefficient	$\hat{\mu }$	Magnetic permeability
*Nu*	Nusselt number	$\mu_{e}$	Magnetic diffusivity
*Pr*	Prandtl number	*μ*	Dynamic viscosity
*q_r_*	Radiative heat flux	υ	Kinematic viscosity
*q_w_*	Heat transfer at wall	*σ*	Stefan-Boltzmann constant
*R*	Radiation parameter	*θ*	Dimensionless temperature
*Re*	Reynolds number	$\tau_{w}$	Wall shear stress
*s*	Nanoparticles shape factor	*ε*	Temperature ratio parameter
*T*	Fluid temperature		**Subscripts**
*T_w_*	Surface temperature	*hnf*	Hybrid nanofluid
*T_∞_*	Ambient temperature	*f*	Base fluid
*u, w*	Velocity components in r-, z- directions.	*w*	Surface condition
*U_w_*	Stretching velocity	∞	Ambient condition
*U_∞_*	Ambient velocity	s_1,_s_2_	Shape factors of Copper and Alumina
